# The multisensory control of sequential actions

**DOI:** 10.1007/s00221-024-06962-0

**Published:** 2024-12-05

**Authors:** Daniel Säfström

**Affiliations:** Department of Medical and Translational Biology, Umeå University, S-901 87 Umeå, Sweden

**Keywords:** Sensorimotor control, Multisensory integration, Sequential actions, Reaching

## Abstract

Many motor tasks are comprised of sequentially linked action phases, as when reaching for, lifting, transporting, and replacing a cup of coffee. During such tasks, discrete visual, auditory and/or haptic feedback are typically associated with mechanical events at the completion of each action phase, as when breaking and subsequently making contact between the cup and the table. An emerging concept is that important sensorimotor control operations, that affect subsequent action phases, are centred on these discrete multisensory events. By predicting sensory feedback at the completion of action phases, and comparing with the actual feedback that arises, task performance can be continuously monitored. If errors are detected, the sensorimotor system can quickly respond with task-protective corrective actions. The aim of this study was to investigate how discrete multisensory feedback at the completion of action phases are used in these control operations. To investigate this question, 42 healthy human participants (both male and female) performed a visually guided sequential reaching task where auxiliary discrete visual, auditory and/or haptic feedback was associated with the completion of action phases. Occasionally however, this feedback was removed in one or two modalities. The results show that although the task was visually guided, its control was critically influenced by discrete auditory and haptic feedback. Multisensory integration effects occurred, that enhanced the corrective actions, when auditory feedback was unexpectedly removed along with haptic or visual feedback. This multisensory enhancement may facilitate the ability to detect errors during sequential actions and amplify task-protective corrective actions.

## Introduction

Manual behaviours, like reaching for different objects, allow humans to interact with their environment, and sensory feedback in different modalities arising from this interaction is critically important for both the planning and execution of manual tasks (Fogassi and Gallese 2004; Sabes 2011; Betti et al. 2021; Camponogara 2023). Most manual tasks are comprised of a series of sequentially linked actions, or action phases, that together accomplish an overall task goal. For instance, the sequential actions of reaching for, grasping, lifting, transporting, and replacing a cup may accomplish the overall goal of drinking coffee. During such tasks, discrete visual, auditory and/or haptic (tactile and proprioceptive) feedback are typically associated with mechanical events at the completion of each action phase, as when breaking and subsequently making contact between the cup and the table. Therefore, many tasks can be characterized as a sequence of discrete sensory events linked to the completion of action phases. These events are typically multisensory: They can be precisely encoded by tactile afferents in the fingers (Sobinov and Bensmaia 2021), often generate sounds from the interaction between objects (Castiello et al. 2010), and gaze fixations are directed to contact events to support visual monitoring of subgoal completions (Johansson et al. 2001). An emerging concept is that these multisensory events are used as “sensorimotor control points” and that important neural control operations that affect subsequent action phases are centred around them (Johansson and Flanagan 2009; van Polanen et al. 2019).

The use of sensory feedback in sequential tasks typically occurs within a largely predictive control strategy, where the feedback is used to monitor the completion of successive task goals. That is, skilful actions involve the capability to predictively specify the efferent control signals (“motor commands”) necessary to complete various tasks, and to predict the sensory consequences of these motor commands (Flanagan et al. 2006). Such a predictive strategy enables that the motor commands for the next phase can be released in anticipation of completion of the current action phase (Säfström and Edin 2008), thereby enabling a smooth linking of action phases (Säfström et al. 2013). By comparing the predicted sensory feedback at goal events with the actual feedback that arises, task performance can be continuously monitored. If errors are detected, that is, discrepancies between predicted and actual feedback, the sensorimotor system can quickly respond with task-protective corrective actions (Johansson and Flanagan 2009; van Polanen 2022; McGarity-Shipley et al. 2023). Moreover, because of the multisensory character of discrete events at the completion of action phases, they enable learning, upholding and adaptation of multimodal sensorimotor correlations that assist the predictive specification of motor commands for future movements (Johansson and Flanagan 2009).

Previous studies have shown that anticipation of sensory “action effects” is important for the acquisition and execution of tasks involving sequences of key presses (Hoffmann et al. 2001; Stöcker & Hoffmann 2004; Stöcker et al. 2003), but few studies have addressed integration of discrete multisensory feedback during sequential actions. A recent study however on bimodal action effects demonstrated that both the initiation and the execution of the action sequence was faster if key presses were associated with audiovisual feedback, as compared with unimodal auditory or visual feedback (Luan et al. 2021). Another study on bimodal discrete feedback showed that force scaling during object lifting was affected when visual feedback was delayed compared with haptic feedback (van Polanen et al. 2019).

The aim of this study is to advance the understanding of multisensory integration during sequential actions, by investigating how discrete trimodal feedback (visual, auditory and haptic) at the completion of action phases are used in control operations where predicted sensory feedback are compared with actual feedback. Sensory events in multiple modalities, that encode different physical properties of mechanical events, may enable a comparison of predicted and actual feedback along different sensory dimensions. Hypothetically, such multisensory comparisons may facilitate error detection and amplify task-protective corrective responses that could contribute to the fulfilment of overall task goals. Also, this multisensory facilitation could resemble the additive or superadditive response enhancement caused by multisensory feedback when detecting stimuli that mediate orientation behaviors (Stein 1998).

To evaluate these hypotheses, and to investigate how multisensory feedback is used to control sequential actions, the participants in this study performed a visually guided reaching task with discrete visual, auditory and/or haptic feedback at the completion of action phases. By sometimes removing this feedback in one or two modalities it was possible to determine how different modalities were used.

## Methods

### Participants

A total of 42 healthy right-handed participants (18 males, 24 females; mean age 25.3 years, range 19–40) with normal vision participated in the study. They received 200 Swedish krona (≈ 18 USD) for participating.

### Experimental design

#### Apparatus

**Fig. 1:**
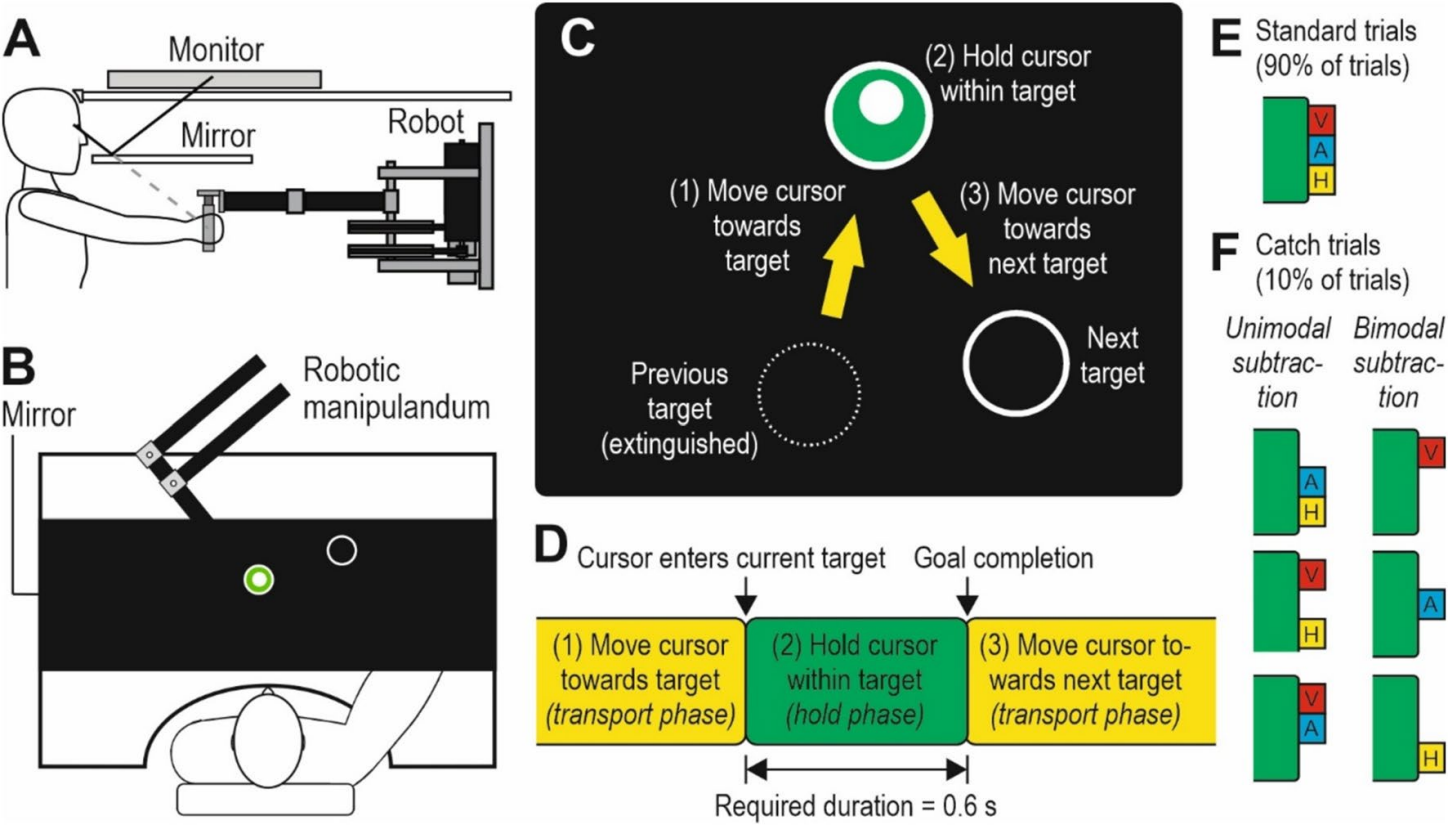
Apparatus, experimental task, and subtractions of sensory feedback. **a** The Kinarm robotic platform (Kinarm End-Point Robot, BKIN Technologies) included a monitor that was visible by means of a mirror. The participants could steer the cursor with their right hand using a handle attached to a robotic arm. **b** The cursor was centred on the handle and was, along with the targets, visible on the otherwise black mirror. The participants could not see their hand or the handle below the mirror. **c** The task was to move a cursor towards a target, to hold the cursor within the target for a required duration, before moving the cursor towards the next target. **d** The task was comprised of sequential action phases of cursor movements (yellow boxes, corresponding to yellow arrows in c) and cursor holding (green box, corresponding to green target in c). The required duration of the hold phase was 0.6 s. **e** In standard trials, synchronous visual (V; a brief flash), auditory (A; a beep) and haptic feedback (H; vibration) indicated goal completion at the end of the required hold duration. **f** In catch trials, that were intermingled between standard trials, the sensory feedback was manipulated by removing feedback in one modality (unimodal subtraction) or two modalities (bimodal subtraction)

The participants sat on a height-adjustable chair in front of a robotic manipulandum platform (Kinarm End-Point Robot, BKIN Technologies; Fig. 1a). This platform includes a virtual reality setup where a monitor is visible by means of a horizontal mirror in front of the participants. The participants forehead rested against a support, and they grasped a handle attached to a robotic arm with their right hand. Using the handle, the participants controlled a cursor that they moved between targets on the screen (Fig. 1b).

The cursor was centred on the handle and was, along with the targets, visible on the otherwise black mirror. The participants could not see their hand or the handle below the mirror. Data were sampled at 1000 Hz. A head-free gaze tracking system allowed registration of eye movements (data from the gaze registrations are not described in this paper).

#### Experimental task

The participants task was to, as quickly as possible, move the cursor towards the sequentially presented targets, to hold the cursor within the target zone for a required duration of time (0.6 s) in order to complete the target, before moving the cursor towards the next target (Fig. 1c). Thus, the task comprised sequential action phases of cursor transport between the targets (transport phase), and cursor holding within the targets (hold phase; Fig. 1d).

Along with the cursor (filled white circle, 0.5 cm radius) there were always two targets visible on the screen: the current target (green circle with 0.9 cm radius, with a 0.1 cm white outline) and the next target (black circle with 0.9 cm radius, with a 0.1 cm white outline). When the cursor entered the next target, it became the current target (that is, it changed colour from black to green). Simultaneously, the previous target was extinguished, and a new next target appeared on an unpredictable location on the screen. Thus, the next target was visible during the hold phase. 64 target locations were distributed equally across the four quadrants of the screen under the constraint that the distance between two successive targets was always 15 cm and the direction from the previous target was uniformly distributed between 0 and 360° (Sailer et al. 2005). The same set of 64 target locations was used for each participant.

During the experimental session the participants were chasing targets during 70 s periods, separated by 8 s periods of rest. Every 5 min, they had a longer period of rest (≈ 1 min) between the periods of target chasing. Each participant completed 1200 trials, after which the experiment ended automatically. The total time for the experiment was about 30 min (all participants then continued with other variations of the task, but data from those experiments are not included in this paper).

#### Instruction to participants

It is known that the purpose, i.e. the specific overall goal, of a motor task can influence the way the task is executed (Buckingham and Donald 2019). To ensure that the participants shared a common notion of the overall goal during the experiments, so that interindividual variability in movement execution could be reduced, they were explicitly told that their goal was to complete as many targets as possible. To motivate the participants, the screen during the rest periods showed the number of completed targets during the previous target chasing period, as well as the “high score” of targets completed during any previous target chasing period.

#### Trial structure

A *trial* consisted of two phases: The *hold phase* started when the cursor entered the target zone and ended at goal completion after 0.6 s (Fig. 1d). The *transport phase* (and the corresponding *transport phase duration*) started at goal completion and ended when the cursor entered the next target zone. The *trial duration* therefore encompassed the duration of the hold phase and the duration of the transport phase. The *clearance time* was defined as the duration between goal completion and cursor exit from the target zone. However, if the cursor exited the target zone < 0.6 s after cursor entry, that is, before goal completion, this was considered a *premature cursor exit*. In such cases the target was not completed, and the participant had to return the cursor to the target zone to complete the required duration, thereby loosing time. Specifically, when a premature cursor exit occurred, the *reentry time* (that is, the duration from the premature cursor exit to cursor reentry into the target zone) was added to the duration of the hold phase, which increased the trial duration.

In summary, three different kinds of events (cursor entry, goal completion and cursor exit) were used to objectively define onset and offset times for the different variables during each trial, and the variables thus reflected the trial structure (for instance, the variable transport phase duration encompassed the transport phase). The time when the cursor entered a target zone (cursor entry) and exited a target zone (cursor exit) was defined as the time the center of the cursor moved across the center of the border that outlined the target on the screen. Notably, cursor entry and exit were directly determined by the spatial position of the cursor relative to the targets, which enabled an exact and unambiguous definition of onset and offset times (for a study of different ways to determine movement onset, see Oostwoud Wijdenes et al. 2014).

#### Discrete multimodal stimuli at goal completion

Besides using vision as primarily guiding the movements, goal completion at the end of the required 0.6 s duration of the hold phase was indicated by discrete auxiliary sensory feedback (Fig. 1 e,f). If the cursor exited the target zone prematurely, i.e. before goal completion, the participant did not obtain feedback and had to return the cursor to the target zone to complete the required duration. The discrete sensory feedback could be visual (target doubled in diameter for 50 ms in a flash-like manner), auditory (a salient 1 kHz beep for 50 ms) and/or haptic (a 10 N load in y-direction for 2 ms, with 4 ms ramp-up and ramp-down periods). Haptic feedback was delivered through vibrations in the robotic handle, and auditory feedback was delivered using soundproof earphones.

In *standard trials* (90% of all trials; i.e. 1080 trials), the discrete feedback consisted of synchronous visual, auditory, and haptic feedback (Fig. 1e). In *catch trials* (10% of all trials; i.e. 120 trials), one or two modalities were *subtracted*, that is, removed (Fig. 1f). The catch trials were intermingled between standard trials, in an unpredictable manner for the participants. Each catch trial was always preceded and succeeded by a standard trial. In total, there were 6 different types of catch trials where either one modality was removed (unimodal subtraction) or two modalities were removed (bimodal subtraction). All 6 types of catch trials occurred an equal number of times (i.e. 20 trials of each type), and the order of presentation of each type was counterbalanced between participants.

### Experimental paradigm

**Fig. 2:**
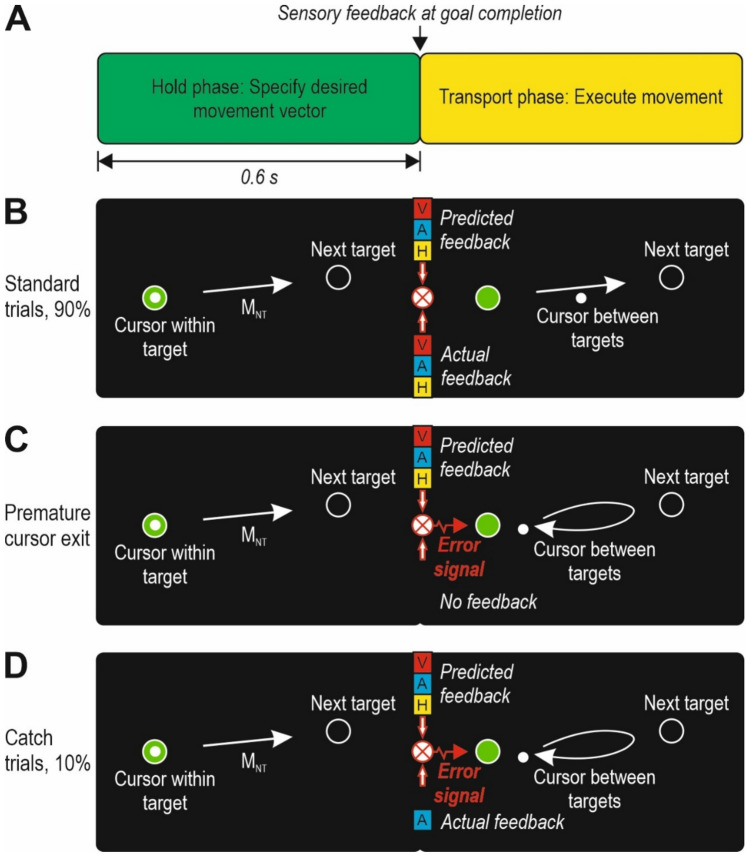
Experimental paradigm. **a** Schematic representation of the hold phase (in green, left side) and the transport phase (in yellow, right side), that were separated by discrete sensory feedback at goal completion. Because the next target was visible already during the hold phase, the desired movement vector for cursor transport towards the next target could be specified in advance of goal completion. The movement vector was executed during the transport phase. **b** In standard trials, goal completion was associated with visual (V), auditory (A), and haptic (H) feedback. Given that 90% of all trials were standard trials, I assumed that the participants would quickly start to predict this trimodal feedback. By comparing the predicted feedback with the actual feedback at goal completion, task progression could be monitored. If there was no discrepancy between the predicted and actual feedback, cursor transport could continue uninterrupted. The white arrow represents the movement vector towards the next target (M_NT_). Because there was no discrepancy between the predicted and the actual feedback, the executed movement (arrow on right side) is similar as the specified movement (left side). **c** If the cursor exited the target zone prematurely, there was no feedback at the time of goal completion, and the cursor had to be returned to the unfinished target to complete it. Thus, the discrepancy between the predicted trimodal feedback and the absence of feedback (i.e. a “trimodal subtraction”) represented an error that had to be corrected. In this case, the executed movement had to be updated and is not similar as the specified movement. **d** I hypothesized that the discrepancy between the predicted trimodal feedback and the feedback in catch trials (where one or two modalities were removed) would also be interpreted as an error and cause an interruption of the cursor path towards the next target, reminiscent of the corrective action due to premature cursor exits. This hypothesized interruption of the cursor path would make the duration of the transport phase prolonged in catch trials as compared with the standard trials. The figure shows an example of a bimodal subtraction, where visual and haptic feedback have been removed

The overall task goal was to complete as many targets as possible, and because the next target was visible already during the hold phase, the participants could improve task performance by specifying the motor commands for the desired movement vector towards the next target in advance of goal completion (Fig. 2a, left side). That is, with such a predictive strategy the motor commands for cursor transport towards the next target could be released in anticipation of goal completion (cf. Säfström et al. 2013), which would facilitate a rapid execution of the desired movement (i.e., the transport phase, Fig. 2a, right side).

In standard trials, goal completion was associated with visual, auditory, and haptic feedback (Fig. 2b). Given that the required hold phase duration was always 0.6 s and that 90% of all trials were standard trials, I assumed that the participants would quickly start to expect trimodal feedback at the time of goal completion. By comparing this predicted feedback with the actual feedback at goal completion, task performance could be monitored through verification of goal completion. If there was no discrepancy between the predicted and actual feedback, cursor transport towards the next target could continue uninterrupted (Fig. 2b, right side).

In trials where the cursor exited the target zone prematurely, that is prior to goal completion, the predicted feedback did not occur (Fig. 2c). In such trials, the cursor had to be returned to the unfinished target to complete it (Fig. 2c, right side). Thus, the discrepancy between the predicted trimodal feedback and the absence of feedback (i.e. a “trimodal subtraction”) represented an error that had to be corrected before the cursor could be transported towards the next target.

In catch trials, one or two modalities were removed from the sensory feedback at goal completion (Fig. 2d). I hypothesized that the discrepancy between the predicted trimodal feedback and the feedback in catch trials would also be interpreted as an error and cause an interruption of the cursor transport towards the next target, or even a return of the cursor towards the completed target (Fig. 2d, right side), reminiscent of the corrective action required because of a premature exit.

### Criteria for multisensory integration

**Fig. 3:**
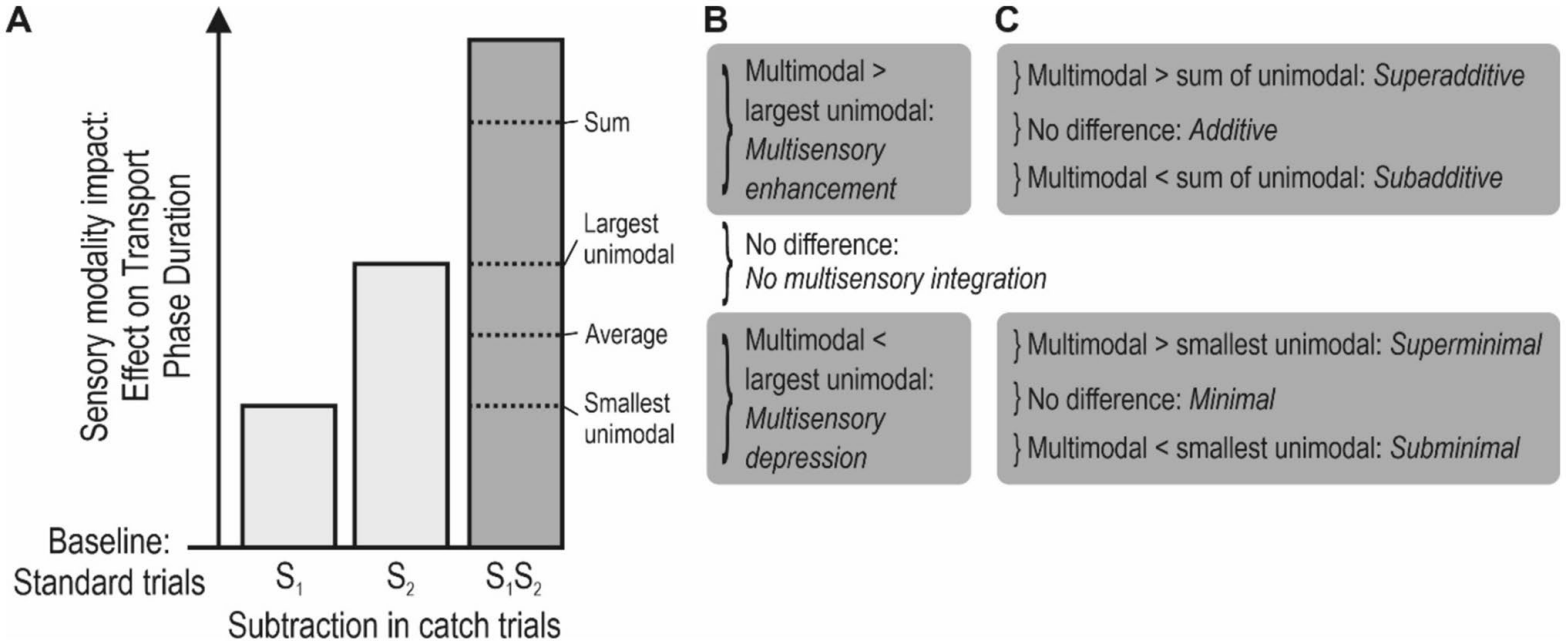
Criteria for multisensory integration. **a** The impact of the subtraction, i.e. the removal, of one or two sensory modalities in the catch trials was operationalized as the *effect on transport phase duration*. The columns denoted S_1_ and S_2_ represent the effect of unimodal subtractions, and the column denoted S_1_S_2_ represent the effect of a bimodal subtraction (where modality S_1_ and modality S_2_ are removed simultaneously). For each participant, the effect on transport phase duration was calculated as the difference in transport phase duration between each type of catch trial (i.e. six values) and the standard trials. For instance, the effect of the bimodal subtraction was calculated as *Transport phase duration*_*S1S2*_*—Transport phase duration*_*Standard trials*_. To explicitly define criteria for multisensory integration effects in catch trials with a bimodal subtraction, I used the effects in catch trials where one modality was removed. The dotted horizontal lines indicate different criteria. **b** Multisensory *enhancement* and multisensory *depression* (because of removed feedback in two modalities) were defined as effects that were statistically significantly larger or smaller, respectively, than the largest unimodal effect (that is, the effect from removing one modality). If there was no significant difference, there was no multisensory integration. **c** Multisensory enhancement was *additive* if the effect equalled the sum of the unimodal effects, that is, if there was no statistically significant difference between them; *superadditive*, if the effect significantly exceeded the sum of the unimodal effects; or *subadditive*, if the effect was significantly smaller than the sum of the unimodal effects. Multisensory depression was *minimal* if the effect equalled the smallest of the unimodal effects, that is, if there was no statistically significant difference between them; *superminimal*, if the effect significantly exceeded the smallest of the unimodal effects; or *subminimal*, if the effect was significantly smaller than the smallest of the unimodal effects

This hypothesized interruption or return of the cursor would presumably make the transport phase duration (the time between goal completion and cursor entry into the next target zone) prolonged in catch trials, as compared with the standard trials. Thus, the transport phase duration during standard trials was defined as the baseline against which the behaviour in the catch trials were compared, and the *effect on transport phase duration* of a catch trial was quantified as the difference compared with the standard trials (Fig. 3a; for a similar baseline cancelling method in fMRI- and ERP-studies, see Miller and D’Esposito 2005; Bonath et al. 2007).

This approach enabled an operationalization of the impact of each modality for monitoring task performance since this would be reflected in the magnitude of the effect on the transport phase duration when that modality was removed. Specifically, if a modality is insignificant for monitoring task performance, I expected that the removal of this modality would not cause a difference in transport phase duration as compared with the standard trials. Conversely, if a modality is significant its removal would cause a prolongation of the transport phase duration.

To explicitly define criteria for *multisensory integration effects* in catch trials where two modalities were removed (bimodal subtractions), I used the effects in catch trials where one modality was removed (unimodal subtractions). Multisensory *enhancement* was defined as an effect that statistically significantly exceeds the largest unimodal effect (Fig. 3b; for a discussion of different criteria for multisensory integration, see Stein et al. 2009). Multisensory *depression* was defined as an effect that is statistically significantly smaller than the largest unimodal effect. Multisensory enhancement can be further classified as *additive*, if the effect equals the sum of the unimodal effects (where “equals” means that there is no statistically significant difference); *superadditive*, if the effect significantly exceeds the sum of the unimodal effects; or *subadditive*, if the effect is significantly smaller than the sum of the unimodal effects (Fig. 3c). Multisensory depression can be further classified as *minimal*, if the effect equals the smallest of the unimodal effects; *superminimal*, if the effect significantly exceeds the smallest of the unimodal effects; or *subminimal*, if the effect is significantly smaller than the smallest of the unimodal effects.

### Statistical analysis

Several repeated measures ANOVAs were made to analyse the data (as detailed in Results). The results of all post hoc comparisons were corrected for cumulative Type 1 errors with the Bonferroni test. A significance level of 0.05 was chosen. The statistical analyses were performed using the software Statistica (version 13, TIBCO Software Inc.).

## Results

In the first part of the Results, I will present data that validate some of the assumptions made in my description of the experimental paradigm (cf. Figure 2). Specifically, I will describe (i) how an overall improvement in task performance was associated with the development of a predictive control strategy; (ii) how premature cursor exits, which were associated with an absence of feedback (i.e. a total trimodal subtraction) at the predicted time of goal completion, caused detrimental effects on task performance; and (iii) how the partial (unimodal or bimodal) subtraction of sensory feedback in catch trials in fact could cause similar corrective actions as those in trials where a premature cursor exit had occurred. In the second part of the Results, I will describe the impact of the sensory subtractions in catch trials, as compared with the standard trials, on the transport phase duration and then analyse those results based on the criteria for multisensory integration (cf. Figure 3).

### Overall learning and development of a predictive control strategy

**Fig. 4:**
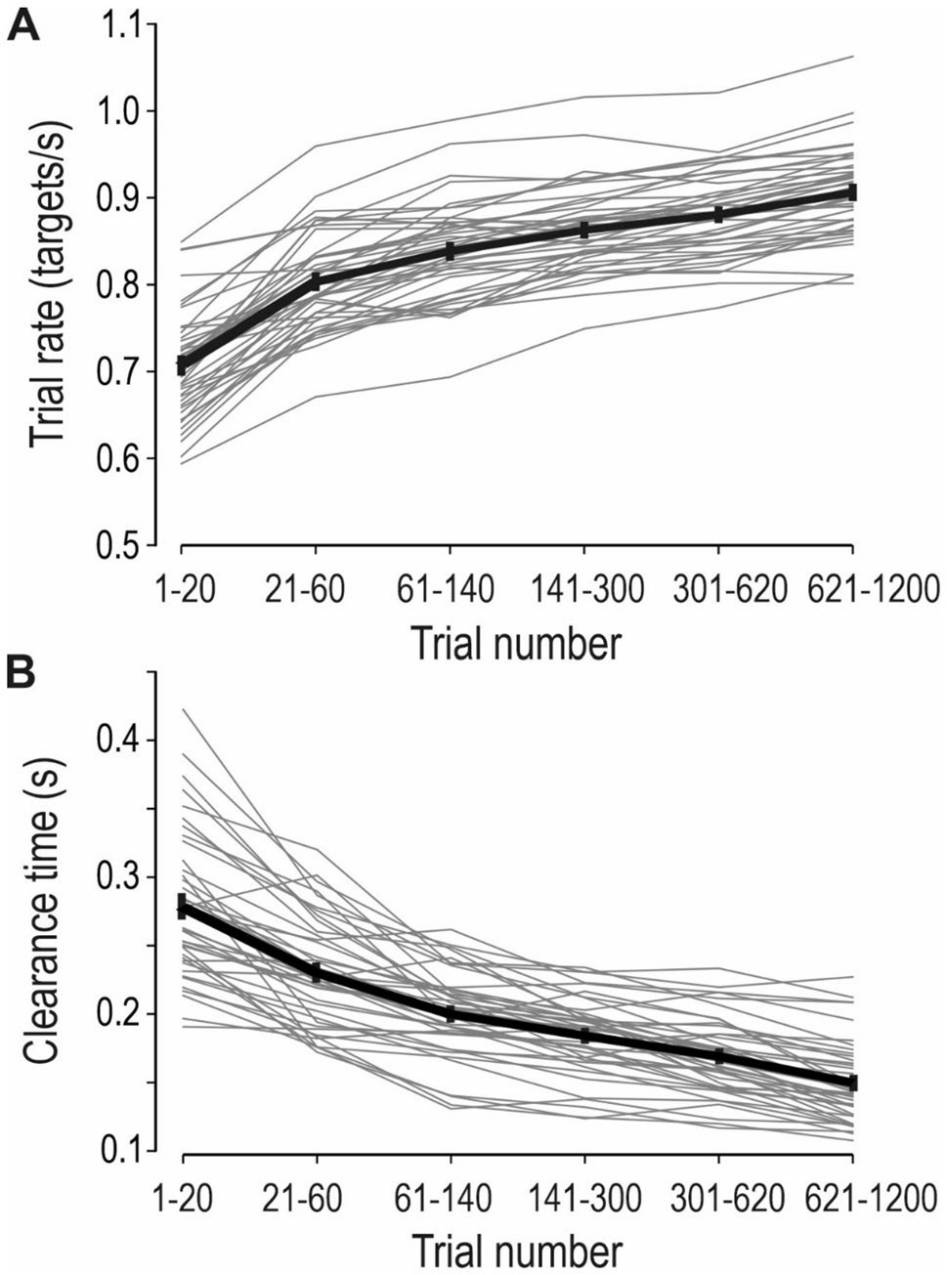
Overall learning and development of a predictive control strategy. **a** To assess overall task performance, the trial rate (i.e., number of targets completed per second) was calculated. The trials were partitioned into six stages that represented approximately equal intervals on a logarithmic scale (trials 1–20, 21–60, 61–140, 141–300, 301–620 and 621–1200). The participants increased their trial rate with practice during the experiment. **b** Clearance time (i.e., time between goal completion and cursor exit from the target zone) during the six stages. The participants significantly reduced the clearance time with learning, which indicate that they attempted to predict the time of goal completion. **a**, **b** Thin gray lines indicate the individual participants, and the thick black line the average across all participants. Error bars represent standard error of the mean (SEM) across participants. Only standard trials are included

The overall task goal for the participants was to complete as many targets as possible. To assess their overall task performance, the *trial rate* was calculated for each trial. The trial rate was defined as ‘the number of trials completed per second’ and was calculated as 1/trial duration (for instance, if the trial duration was 2 s, that would correspond to a trial rate of 0.5 trials/second). To facilitate data analysis, the trials were partitioned into six stages that represented approximately equal intervals on a logarithmic scale. The stages were chosen because they could capture the changes in performance with learning (trials 1–20, 21–60, 61–140, 141–300, 301–620 and 621–1200). The participants significantly improved their trial rate with practice during the ≈ 1200 trials performed by each participant (Fig. 4a; F(5,205) = 322.9, p < 0.0001).

Learning was fastest in the beginning, and then gradually slowed. However, the learning process was protracted and continued for several hundreds of trials, as post hoc analyses revealed significant differences in trial rate between all six stages (p < 0.05 in all cases). As such, the participants seem to have adhered throughout the experiment to the explicit instruction to complete as many targets as possible.

Early in the experiment, when the task was novel to the participants, I expected that the feedback at goal completion would reactively trigger cursor transport towards the next target and that the *clearance time* (the duration between goal completion and cursor exit from the target zone) would reflect the reaction time for starting the movement. On average, the cursor exited the target 0.28 s after goal completion during trials 1 −20, which is similar to reaction times in previous studies on sequential motor tasks (Säfström et al. 2013). However, given that the required hold phase duration was always 0.6 s and that 90% of all trials were standard trials, I assumed that the participants would quickly start to predict the sensory feedback at the time of goal completion (cf. Figure 2b). This would enable them to release the motor commands for the specified cursor movement in anticipation of goal completion, thereby increasing the trial rate. As expected given such a predictive strategy, the participants significantly reduced the clearance time with learning (Fig. 4b; F(5,205) = 189.1, p < 0.0001). In parallel with the increase in trial rate, the clearance time decreased quickly in the beginning of the experiment and there was also protracted learning all through the experiment, as post hoc analyses revealed significant differences in clearance time between all six stages (p < 0.05 in all cases). I conclude that the participants improved their overall task performance with practice, and that this was associated with the development of a predictive control strategy.

### Premature cursor exits

**Fig. 5:**
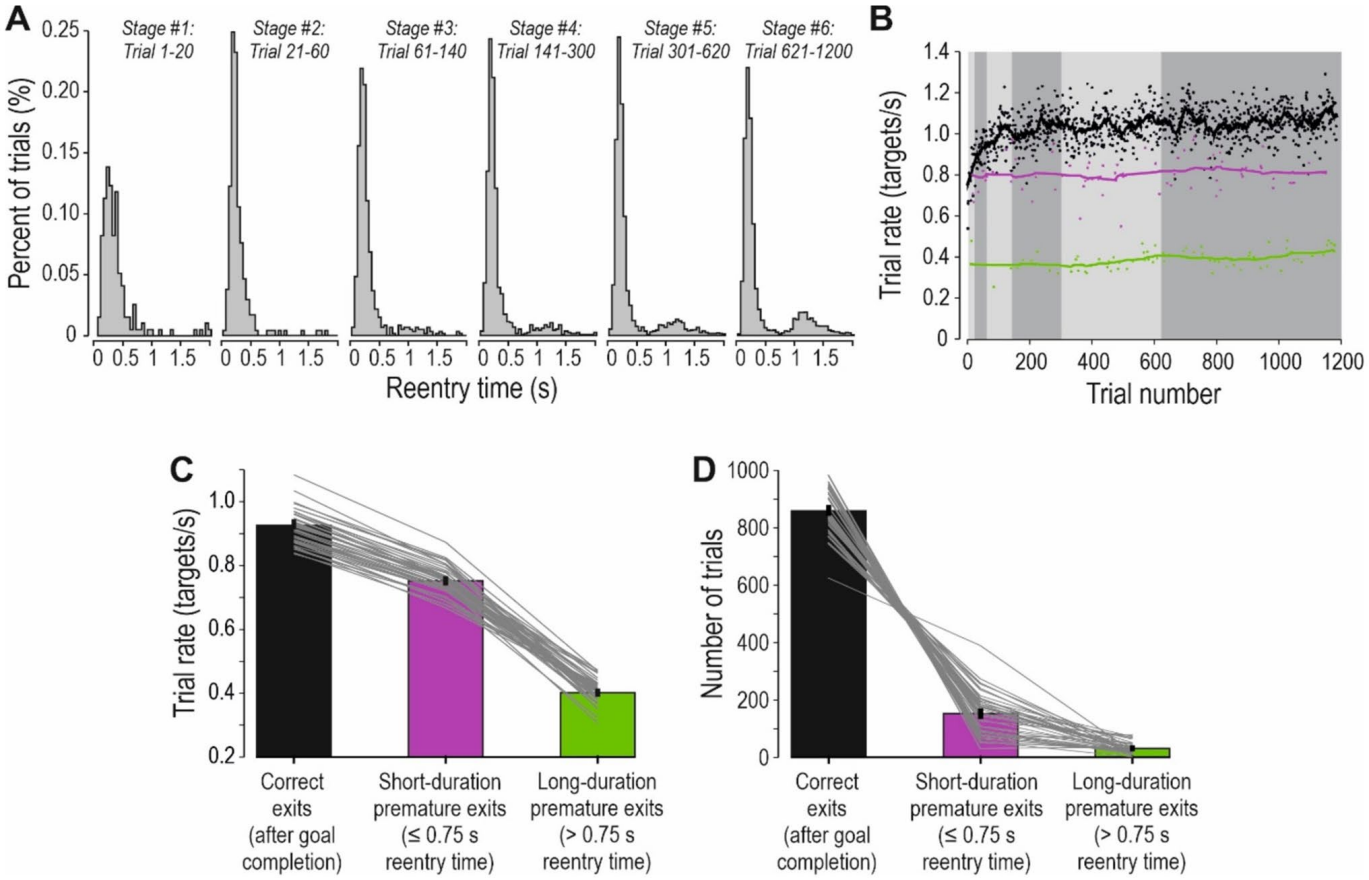
Premature cursor exits. **a** The distribution of reentry times (i.e., the time from the premature cursor exit to cursor reentry into the target zone) during the six stages. With learning, reentry times with longer duration (≈ 1–1.5 s) became more common, presumably because the participants developed their predictive control strategy where they intentionally aimed at the next target, and therefore had to return a longer distance. **b** The trial rate during the experiment for one representative participant. The dots indicate each standard trial. Black dots indicate trials where the cursor exited after goal completion (“correct exits”). Purple dots indicate trials where the cursor exited prematurely and was returned to the target zone within ≤ 0.75 s (“short-duration premature exits”). Green dots indicate trials where the cursor exited prematurely and was returned to the target zone after > 0.75 s (“long-duration premature exits”). The lines represent moving averages across ± 10 trials. The six stages are delineated by the light and dark gray areas. **c** Columns indicate average trial rate during trials where the cursor exited correctly (black column), short-duration premature exits (purple column) and long-duration premature exits (green column). Thin gray lines indicate the individual participants. Error bars represent standard error of the mean (SEM) across participants. **d** Columns indicate the average number of trials in each category (same format as in c). **a**–**d** Only standard trials are included. Only trials where the reentry time was ≥ 0.01 s were categorized as premature exits

However, if the cursor exited the target zone too early, the feedback at goal completion did not occur and the participants had to return the cursor to the target zone to complete the required duration (cf. Figure 2c). Thus, a premature cursor exit resulted in time losses because of the additional time spent outside the target zone in these trials. To analyse these time losses, the *reentry times* (that is, the duration from the premature cursor exit to cursor reentry into the target zone) were calculated. Early in the experiment (stage 1–2), the reentry times almost entirely had a short duration (≈ 0–0.5 s; Fig. 5a).

Presumably, these short-duration premature exits occurred because the cursor accidentally overshot the target or mistakenly slipped from the target zone, and therefore quickly could be moved back. With learning, the time losses more frequently had a longer duration (≈ 1–1.5 s), and the distribution of reentry times became bimodal. These long-duration premature exits presumably occurred because the participants developed a predictive control strategy where the motor commands for cursor movement towards the next target were released in anticipation of goal completion. That is, they intentionally aimed at the next target but exited too early, and therefore had to return a longer distance.

To elucidate the effects on task performance caused by premature exits, their effects on the trial rate were analysed. As expected, long-duration premature exits (trials where the re-entry time was > 0.75 s) considerably decreased the trial rate, as compared with trials where the cursor exited correctly (Fig. 5b). Short-duration premature exits (re-entry time ≤ 0.75 s) also decreased the trial rate, but more moderately. Overall, there was a significant difference in trial rate between these three different categories of trials (Fig. 5c; F(2,82) = 2636.3, p < 0.0001). Post hoc analyses showed that, as compared with correct exit trials, the trial rate was significantly decreased in both categories of premature exit trials (p < 0.0001 in both cases). The trial rate was also significantly decreased in trials with long-duration premature exits, as compared with trials with short-duration premature exits (p < 0.0001). Thus, trials where the participants intentionally moved the cursor towards the next target but mistakenly exited too early (long-duration premature exits), resulted in large errors in task performance.

Given this reduction in trial rate, it was beneficial for participants to exit correctly. Overall, there was a significant difference in how often the three different categories of exits occurred (Fig. 5d; F(2,82) = 1414.5, p < 0.0001). Post hoc analyses showed that correct exits were significantly more common than short- or long-duration premature exits (p < 0.0001 in both cases). The long-duration premature exits were also significantly fewer than those with short-duration (p < 0.001). I conclude that premature cursor exits were relatively uncommon as compared with correct exits, and particularly long-duration premature exits had large detrimental effects on task performance.

### Exemplary recordings of corrective actions

When a premature cursor exit occurred, there was no sensory feedback at the predicted time for goal completion. In the experimental paradigm, it was assumed that a lack of sensory feedback in one or two modalities in the catch trials likewise would generate an error signal that would initiate a corrective action, where the participants interrupted the cursor movement towards the next target or moved it back towards the completed target (cf. Figure 2d).

**Fig. 6:**
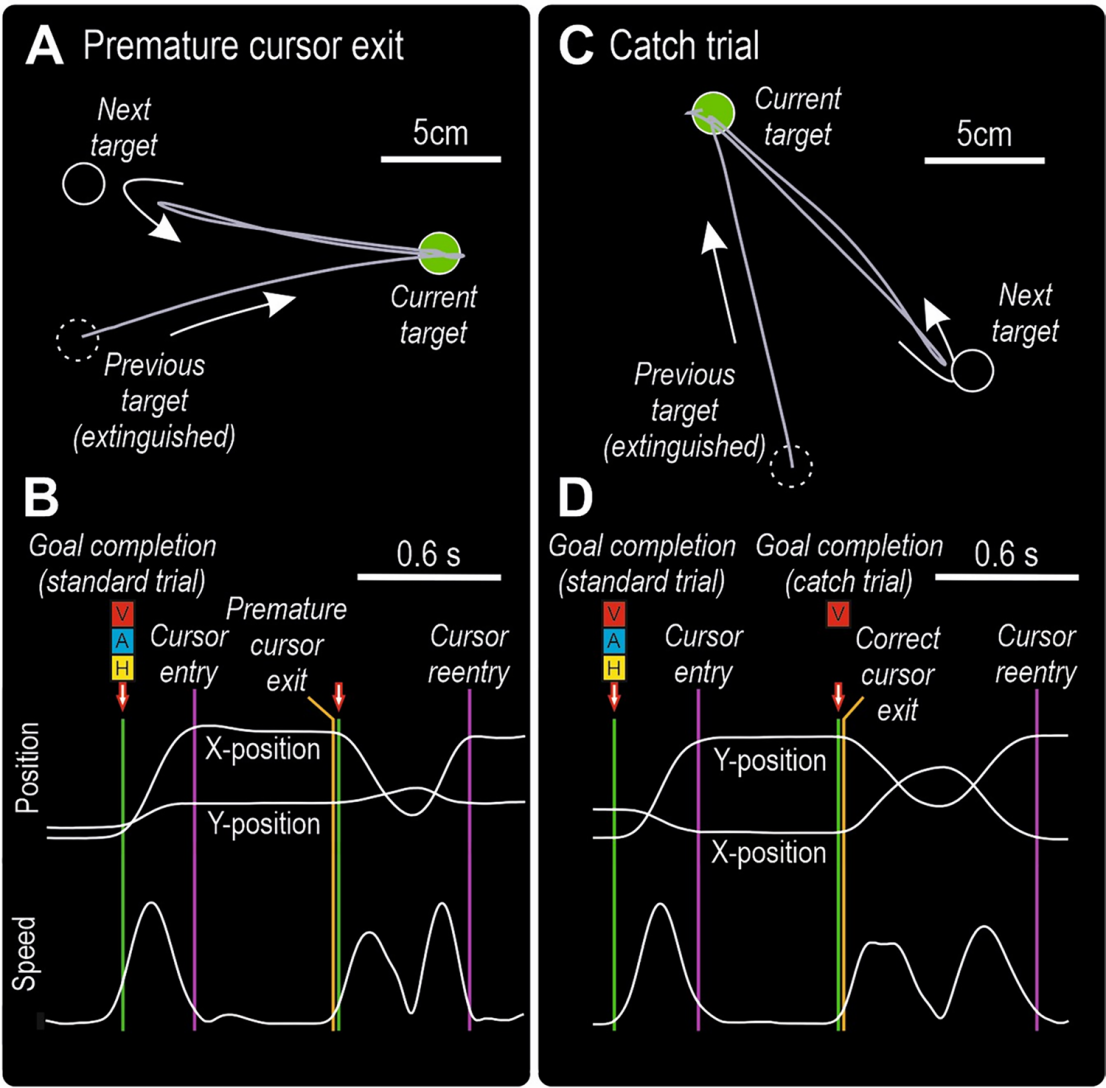
Recordings of exemplary trials. **a** The cursor exited correctly from the previous target and was moved along a straight path to the current target. The cursor exited however prematurely from the current target and was transported to the vicinity of the next target before it was moved back to the current target. **b** Cursor position in the x and y plane, along with cursor tangential velocity, as a function of time for the same trial as shown in a. **c** The cursor exited correctly from the previous target and was moved along a straight path to the current target. The cursor exited correctly from the current target, but the transport towards the next target was interrupted, and the cursor was moved back to the current target. **d** Cursor position and tangential velocity for the same trial as shown in c. Notably, goal completion was indicated by visual feedback only. Even though the cursor exited correctly, the cursor was moved back as if a premature cursor exit had occurred. **a**, **c** The lines indicate the position of the cursor on the screen, and the circles indicate target zones. The arrows indicate the direction of the cursor. **b**, **d** The vertical green lines indicate the end of the required hold phase duration. If the cursor remained in the target at this time point, goal completion was associated with visual (V), auditory (A) and/or haptic (H) feedback. The vertical purple lines indicate the time of cursor entry and reentry into the current target zone. The vertical orange lines indicate cursor exit from the current target. Notably, if the cursor exited prematurely, as in b, no feedback was delivered at the time of goal completion. **a**–**d** The exemplary trials are from the same representative participant as the data in Fig. 5b

Recordings of the cursor path when premature exits occurred showed that the cursor typically was transported to the vicinity of the next target before it quickly was returned to the uncompleted target (Fig. 6a). Consequently, these corrective actions had a characteristic velocity profile, with two adjacent peaks between the premature cursor exit and cursor reentry (Fig. 6b). Recordings of catch trials showed that both the cursor path (Fig. 6c) and the velocity profile (Fig. 6d) could be very similar as when a premature cursor exit had occurred. I conclude that the partial subtraction of sensory feedback in catch trials could generate error signals and corrective actions, akin to those caused by the total lack of feedback due to premature exits.

### The effect of sensory subtractions in catch trials

**Fig. 7:**
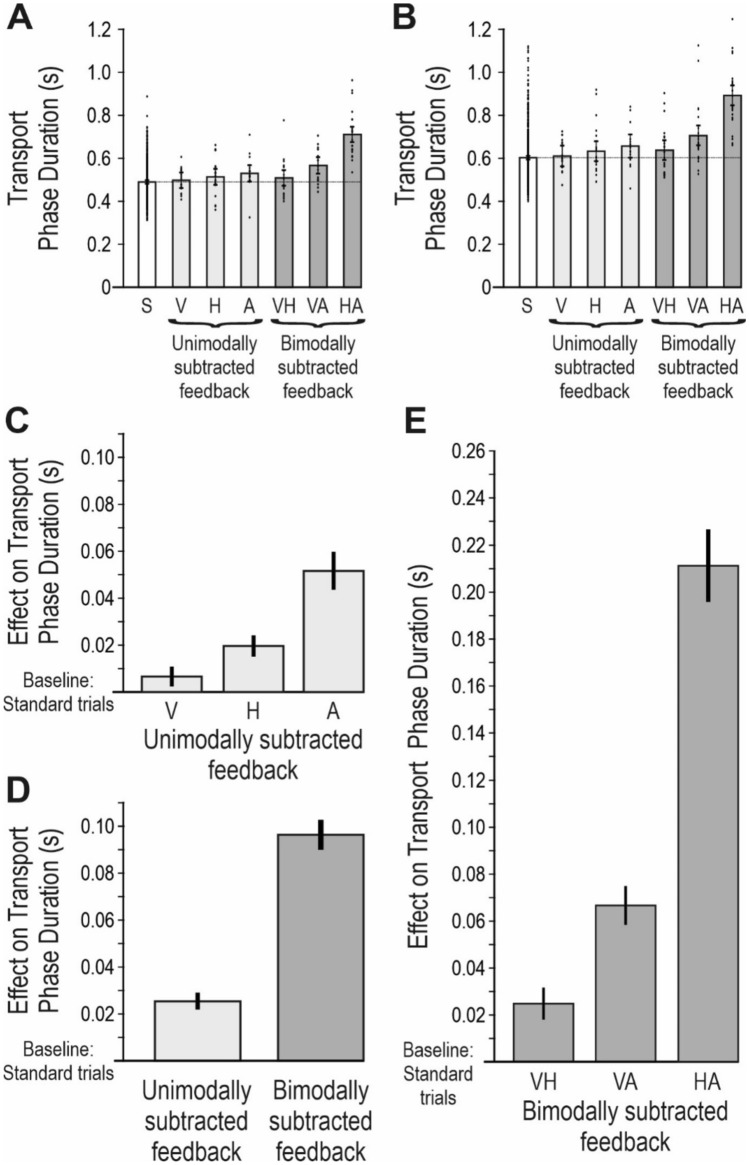
The effect of sensory subtractions in catch trials. **a** The columns show the average transport phase duration, for one representative participant, in standard trials (S) and in each type of catch trial. Standard trials are depicted with white columns, unimodal subtractions are depicted with light gray columns and bimodal subtractions with dark gray columns. The dots indicate individual trials. Error bars represent 0.95 confidence intervals. The horizontal dotted line represent the standard trials/baseline for this participant. **b** Transport phase duration for another participant (same format as in a). Note that the average transport phase duration for each type of trial is longer, and that the level of the baseline is higher, than in a. However, the pattern of the increased duration in different catch trials, as compared with the baseline, is similar as in a. **c**–**e** The columns show the effects on transport phase duration in catch trials, as compared with the standard trials (the baseline). All columns represent average values across all participants. Error bars represent standard error of the mean (SEM) across all participants. **c** The effect because of subtracted visual, haptic or auditory feedback. **d** The average effects caused by unimodal and bimodal subtractions. **e** The effect because of subtracted visual-haptic, visual-auditory or haptic-auditory feedback. **a**–**e** In the catch trials, visual (V), haptic (H), auditory (A), visual-haptic (VH), visual-auditory (VA) or haptic-auditory (HA) feedback was/were subtracted from the feedback at goal completion, and all abbreviations (for instance “V” or “HA”) indicate which modality/modalities that has/have been *removed* from the feedback at goal completion

I hypothesized that the effect of corrective actions in catch trials would be an increase in the duration of the succeeding transport phase, and indeed, it was slightly prolonged in the catch trials as compared with the standard trials (Fig. 7a,b).

However, the participants sometimes differed in their average transport phase duration for each type of trial (i.e. their “baseline” duration (Fig. 7a,b). Therefore, the effect on transport phase duration in catch trials was obtained by subtracting the average transport phase duration during standard trials.

#### Unimodal subtractions

To investigate the effect of the unimodal subtractions, the difference in transport phase duration between standard trials, and trials with subtracted visual, haptic, or auditory feedback was analysed (Fig. 7c).

There was indeed a significant main effect between conditions (F(3,123) = 29.6, p < 0.0001). Post hoc analyses revealed that the duration of the transport phase was significantly increased as compared with baseline when haptic feedback (p < 0.01) or auditory feedback (p < 0.0001) was removed, and significantly longer when auditory feedback was removed, as compared with haptic feedback (p < 0.0001). There was no significant difference between baseline and trials where visual feedback was removed. In conclusion, the subtraction of auditory feedback had a larger impact than the subtraction of haptic feedback, which had a larger impact than the subtraction of visual feedback.

#### Unimodal versus bimodal subtractions

Then the average effects on the transport phase duration caused by unimodal and bimodal subtractions were compared. If the effect of bimodal subtractions reflected the average effect of its constituent unimodal subtractions, there would be no significant difference between them (see Fig. 3a). There was however a large difference, where the average effect from bimodal subtractions was almost 4 times larger than the effect from unimodal subtractions (F(1,41) = 227.5, p < 0.0001; Fig. 7d).

#### Bimodal subtractions

Next the effects on transport phase duration because of the different bimodal subtractions were analysed (Fig. 7e). There was a significant main effect when these three different conditions and the standard trials were compared (F(3,123) = 201.1, p < 0.0001). In post hoc analyses, the transport phase duration was significantly increased as compared with the baseline when visual-auditory or haptic-auditory feedback were removed (p < 0.0001 in both cases). The duration was also significantly longer when haptic-auditory feedback was removed, as compared with visual-auditory feedback (p < 0.0001), and when visual-auditory feedback was removed, as compared with visual-haptic feedback (p < 0.0005). There was no significant difference, compared with baseline, when visual-haptic feedback was removed (p = 0.056). In conclusion, the subtraction of auditory feedback contributed more to the effect of the bimodal subtractions than the subtraction of haptic feedback, which contributed more than the subtraction of visual feedback, that is, [VA + HA] > [VH + HA] > [VH + VA].

### Multisensory integration

To analyse if multisensory integration effects occurred when two modalities were subtracted, the effect of each bimodal subtraction was compared with the largest unimodal effect to determine if there were multisensory enhancement and with the sum of the two unimodal effects to determine if there were superadditive, additive or subadditive effects (Fig. 3b,c). The smallest unimodal effects were not included because the effects of bimodal subtractions were never smaller than the largest unimodal effects, that is, multisensory depression never occurred.

#### Visual-haptic subtraction: No multisensory integration

**Fig. 8:**
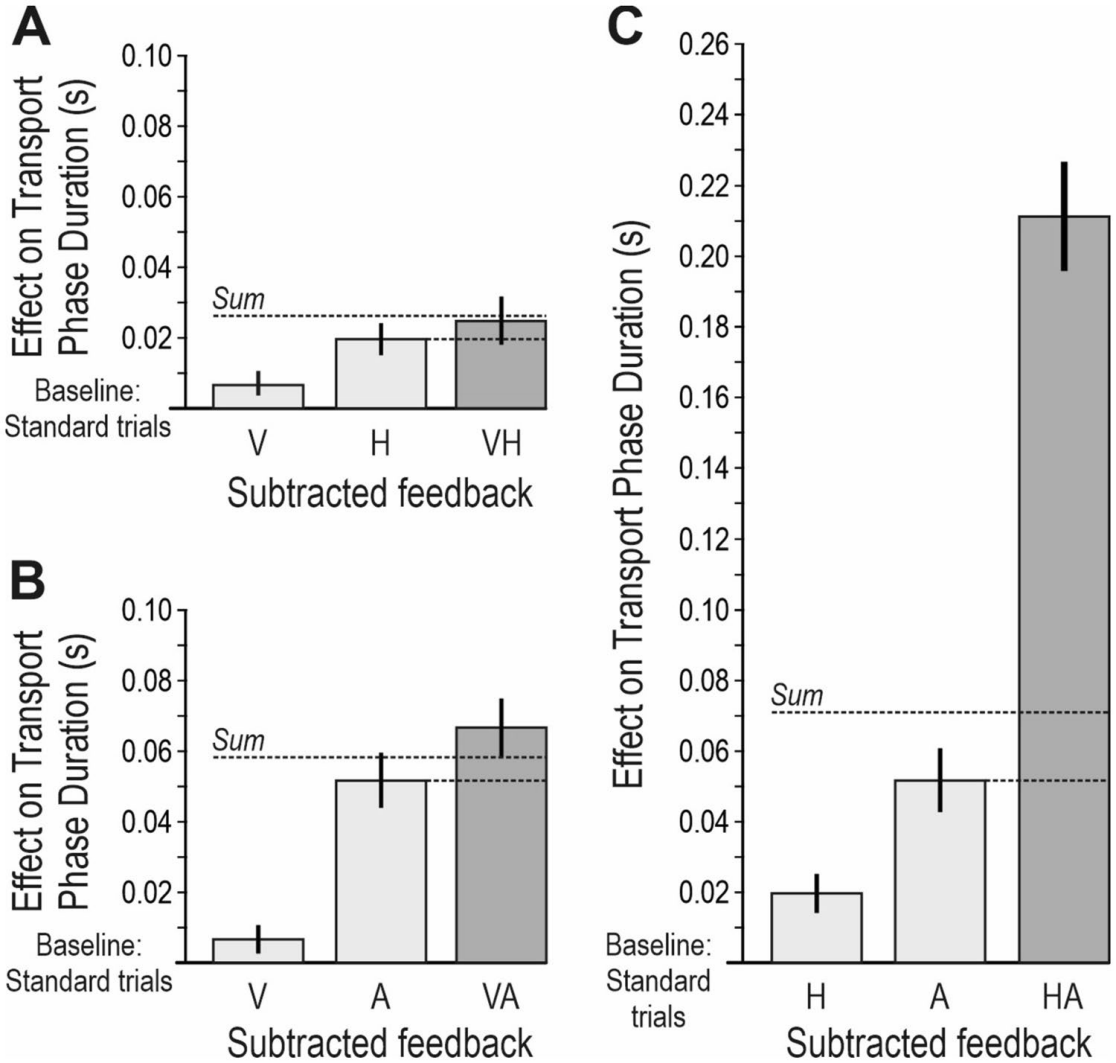
Multisensory integration. **a**–**c** The columns show the effects on transport phase duration in catch trials, as compared with the standard trials (the baseline). **a** The effect because of subtracted visual (V), haptic (H) or visual-haptic (VH) feedback. There was no significant multisensory integration between visual and haptic feedback. **b** The effect because of subtracted visual (V), auditory (A) or visual-auditory (VA) feedback. There was additive multisensory enhancement between visual and auditory feedback. **c** The effect because of subtracted haptic (H), auditory (A) or haptic-auditory (HA) feedback. There was superadditive multisensory enhancement between haptic and auditory feedback. **a**–**c** All columns represent average effects across all participants. Unimodal subtractions are depicted as light gray columns and bimodal subtractions as dark gray columns. Error bars represent standard error of the mean (SEM) across all participants. The horizontal dotted lines represent the calculated summed effect of the two unimodal subtractions and the largest unimodal effect, respectively. Notably, all abbreviations (for instance “V” or “HA”) indicate which modality/modalities that has/have been removed from the feedback at goal completion

There was no statistically significant difference in effect on the transport phase duration between the bimodal visual-haptic subtraction, the unimodal haptic subtraction, and the sum of the unimodal visual and haptic subtractions (Fig. 8a; F(2,82) = 1.1, p = 0.35). Thus, there was no significant multisensory integration between visual and haptic feedback.

#### Visual-auditory subtraction: Additive multisensory enhancement

There was a significant difference when comparing the bimodal visual-auditory subtraction, the unimodal auditory subtraction, and the sum of the unimodal visual and auditory subtractions (Fig. 8b; F(2,82) = 3.5, p < 0.05). In post hoc analyses, the effect on the transport phase duration was significantly larger because of the visual-auditory subtraction as compared with the unimodal auditory subtraction (p < 0.05), which entails that multisensory enhancement occurred. There was however no difference between the visual-auditory subtraction and the sum of the unimodal visual and auditory subtractions (p = 0.42). Therefore, I conclude that the multisensory enhancement between visual and auditory feedback was additive.

#### Haptic-auditory subtraction: Superadditive multisensory enhancement

There was also a significant difference when comparing the haptic-auditory subtraction, the unimodal auditory subtraction, and the sum of the unimodal haptic and auditory subtractions (Fig. 8c; F(2,82) = 213.4, p < 0.0001). Post hoc analyses showed that the effect on the transport phase duration was significantly larger because of the haptic-auditory subtraction as compared with the unimodal auditory subtraction (p < 0.0001). Therefore, multisensory enhancement occurred. The effect was also significantly larger from the haptic-auditory subtraction as compared with the sum of the unimodal haptic and auditory subtractions (p < 0.0001). Therefore, I conclude that there was superadditive multisensory enhancement between haptic and auditory feedback.

## Discussion

The aim of this study was to advance the understanding of multisensory integration during sequential actions, by investigating how discrete trimodal feedback at the completion of action phases are used in control operations where predicted sensory feedback are compared with actual feedback. The results show that although the task was visually guided, its control was critically influenced by discrete auditory and haptic feedback. In accordance with the hypotheses, multisensory integration effects occurred, that enhanced task-protective corrective actions, when auditory feedback was unexpectedly removed along with haptic or visual feedback. The visual-auditory subtraction produced an additive enhancement, whereas the haptic-auditory subtraction produced a superadditive enhancement.

### Multisensory enhancement

This multisensory enhancement may facilitate the ability to detect errors during task performance and amplify the corresponding task-protective corrective actions. These findings add to previous discoveries that multisensory integration can facilitate functionally advantageous behaviours. For instance, multimodal stimuli generate enhanced neuronal responses, as compared to unimodal stimuli, in the superior colliculus (Meredith and Stein 1986), where individual neurons continuously transform unimodal stimuli to an integrated multisensory response (Miller et al. 2017). This increases the probability of detecting multimodal stimuli, and enhance the corresponding orienting behaviors (Stein 1998; Stein et al. 2009). Other multisensory neurons, within a frontoparietal network, contribute to a representation of peripersonal space and their response to tactile stimulation can be enhanced by simultaneous auditory or visual stimulation (Serino 2019). This multisensory enhancement may facilitate precise and adaptable goal-directed movements, defensive behaviors and predictions about the sensorimotor consequences of interactions with objects (Graziano and Cooke 2006; Brozzoli et al. 2012). Relatedly, visual discrimination performance at the target location for reaching movements can be enhanced by spatiotemporally aligned auditory feedback (Elshout et al. 2020), and both the initiation and the execution of sequential key presses is faster if each key press is associated with bimodal audiovisual feedback, as compared with unimodal auditory or visual feedback (Luan et al. 2021). Multisensory enhancement also facilitates manual reactions times, which are shorter when visual, auditory, and haptic stimuli are presented together (trimodal stimuli) as compared with bimodal combinations, which in turn are shorter than reactions to unimodal stimuli (Diederich and Colonius 2004).

### The experimental paradigm

The task was designed to require both spatial and temporal motor control (Georgopoulos 2002; Brass and Haggard 2008; Hoffstaedter et al. 2013). This control involved specifying an appropriate action (a desired movement vector), which can be conceptualized as a process whereby “motor commands” are specified to activate muscles that will move the hand to the desired target location (Kawato and Wolpert 1998). However, within a different theoretical framework, this process can be conceptualized as a shift of the “referent hand position”, that will drive movement through a process whereby the difference between the current and the referent position is minimized (Latash et al. 2010; Yang and Feldman 2010).

The task also captures several other key features of natural sequential tasks: It has an explicit overall goal which is accomplished by a series of sequentially linked action phases; each phase needs to be completed before the next phase can be executed; and launching the next action phase before the current phase is completed results in performance errors and substantial delays in overall task completion time (Flanagan et al. 2006). Additionally, discrete multimodal sensory events mark the completion of action phases, which is akin to many natural tasks, such as making tea in a kitchen (Land et al. 2006), that are performed in “rich” multimodal environments where discrete visual, auditory and haptic feedback can be used to monitor completions of action phases, such as making or breaking contact between different objects. Finally, this task involves a sequence of goal-directed reaching-movements, whereas many studies of motor control have examined single actions, such as moving the hand between two positions (Shadmehr et al. 2010; Wolpert and Flanagan 2016) or sequential actions involving repeated finger-press responses (Bortoletto and Cunnington 2010; Luan et al. 2021; Stefanescu et al. 2013; Wiestler and Diedrichsen 2013). Of course, I acknowledge that this laboratory task differs is some respects from many natural tasks. For example, many natural tasks do not include action phases with an externally specified duration, or a predetermined sequence of movements. Natural tasks are typically less constrained and involve decisions between different potential movements (Michalski et al. 2020).

### Action-phase controllers and visual control of sequential tasks

The overall task goal was to complete as many targets as possible, which was achieved by successively performing smaller actions (“transport phase”, “hold phase”) which themselves can be considered as “motor primitives” (Flash and Hochner 2005) or as “functional units” with their own subgoals that nest together to form the overall activity (van Dijk et al. 2017). Relatedly, implementation of an overall task can be conceptualized as the selection and execution of appropriate “action-phase controllers”, which are a “control policies” that uses sensory information to generate motor commands in order to attain sensory goals (Johansson and Flanagan 2009). The sensory information needed to generate accurate motor commands includes information about the current state of the motor apparatus and the environment. Information about the motor apparatus may have been inferred from the visual position of the cursor which indicated hand position. Moreover, given that the participants could not see their hand, it may also have involved proprioceptive feedback about the configuration of the elbow and shoulder joint.

Visual feedback about the spatial position of the target was essential, since the desired movement vector was specified in a visual reference frame (Fig. 2). During visually guided movements, the action‑phase controllers inform task- and phase-specific eye movements that supports the planning and control of hand actions (Johansson and Flanagan 2009; Säfström et al. 2014). For example, gaze fixations are generally directed at discrete positions where contact events subsequently take place, such as sites where fingertips are placed on objects (Johansson et al. 2001). Typically, gaze exits from these positions coincide with the contact event, suggesting that subgoal completions are visually monitored. Using the referent control framework, it has been suggested that changes in the referent eye position towards a specified target, which elicits a gaze shift, can be rescaled to a corresponding referent hand position, which is then transmitted to motoneurons of arm muscles that drive hand movements towards the target (Zhang et al. 2022). Visual feedback can also guide interceptive movements towards non-stationary targets with high temporal precision, as when reaching towards a computer screen to tap on moving targets (Brenner and Smeets 2015).

### Auditory and haptic encoding of temporal events

Although the task was visually guided, task control was mainly influenced by the removal of discrete auditory and/or haptic feedback. This is consistent with findings that, even if vision provides veridical information about an object, incongruent haptic feedback can influence both the perceptual estimation of object size and grasping actions towards the object (Pettypiece et al. 2010). One explanation for the significant impact of haptic and auditory subtractions is plausible given that goal completion indicated a temporal event and that the sensorimotor system primarily predicts the timing of sensory feedback (Säfström and Edin 2008): Specifically, previous studies have shown that haptic feedback is important for encoding the temporal aspect of movements (Aschersleben et al. 2001), and that audition generally is superior to vision for temporal processing (Aschersleben and Bertelson 2003), even during conditions where auditory feedback is less salient or ignored (Ortega et al. 2014). Therefore, discrete haptic and auditory feedback may have been integrated into the predicted feedback at goal completion because these modalities provide accurate temporal estimates.

The finding that the removal of temporally accurate auditory feedback influenced error detection most, followed by haptic feedback, is in line with findings that when auditory-tactile feedback about temporal events are asynchronous, movements are predominantly influenced by the auditory stimuli (Roy et al. 2017). It is also consistent with the concept that multisensory integration is regulated such that each modality is weighted in relation with its accuracy, whereby the integrated estimate represents the most reliable combination of the unimodal inputs (Ernst and Banks 2002; Helbig and Ernst 2007), and the concept that the weighting of sensory information during movement planning can be regulated to satisfy task requirements, as when haptic feedback is weighted more when it crucially contributes to avoiding errors during grasping movements (Säfström and Edin 2004).

### Attention and explicit expectations of sensory feedback

Previous research has shown that reaction times are shorter to a stimulus in an expected modality (visual, auditory, or haptic), as compared to when the stimulus is unexpected (Spence et al. 2001). Relatedly, participants respond quicker to haptic targets when they are preceded by another haptic target than when they are preceded by a visual or an auditory target. Such attentional effects may have contributed to the finding that the transport phase duration was shorter during standard trials (where the feedback was expected), as compared with the catch trials (unexpected). However, it does not explain the difference between different kinds of catch trials, since they all occurred a similar number of times and their presentation was counterbalanced between participants, and they therefore were equally probable. Furthermore, even if the participants would have expected the catch trials, it is unlikely that this expectation per se would have affected their performance, since that although the availability of sensory feedback affects motor performance (larger aperture during grasping if visual feedback is absent), there is no difference between conditions where this availability varies randomly or predictably. That is, explicit knowledge of the availability does not affect movement planning (Whitwell et al. 2008).

## Conclusions

This study expands the understanding of how discrete multimodal feedback associated with the completion of action phases are used as sensorimotor control points during sequential actions. Specifically, discrete auditory and haptic feedback enhance the ability to detect and respond to performance errors. Understanding the principles that regulate the multisensory control of sequential actions may have importance for the broader understanding of motor planning, control and learning, and may also inspire the development of techniques for augmentation or rehabilitation of sensory functions. This behavioural study does not provide direct knowledge about the neural mechanisms or brain activation patterns engaged in the control of sequential actions, nor does it unravel the presumably intricate causal structure involved in the coupling between sensory stimuli and motor output (Turvey and Sheya 2017). Additional studies, using for instance functional brain imaging, are needed to elucidate these issues.

## Data Availability

The datasets generated during and/or analysed during the current study are available from the author on reasonable request.

## References

[CR1] Aschersleben G, Bertelson P (2003) Temporal ventriloquism: crossmodal interaction on the time dimension. 2. Evidence from sensorimotor synchronization. Int J Psychophysiol 50:157–163. 10.1016/S0167-8760(03)00131-4 14511843 10.1016/s0167-8760(03)00131-4

[CR2] Aschersleben G, Gehrke J, Prinz W (2001) Tapping with peripheral nerve block. A role for tactile feedback in the timing of movements. Exp Brain Res 136:331–339. 10.1007/s002210000562 11243475 10.1007/s002210000562

[CR3] Betti S, Castiello U, Begliomini C (2021) Reach-to-grasp: a multisensory experience. Front Psychol 12:614471. 10.3389/fpsyg.2021.614471 33633644 10.3389/fpsyg.2021.614471 PMC7900505

[CR4] Bonath B, Noesselt T, Martinez A, Mishra J, Schwiecker K, Heinze HJ, Hillyard SA (2007) Neural basis of the ventriloquist illusion. Curr Biol 17:1697–1703. 10.1016/j.cub.2007.08.050 17884498 10.1016/j.cub.2007.08.050

[CR5] Bortoletto M, Cunnington R (2010) Motor timing and motor sequencing contribute differently to the preparation for voluntary movement. Neuroimage 49:3338–3348. 10.1016/j.neuroimage.2009.11.048 19945535 10.1016/j.neuroimage.2009.11.048

[CR6] Brass M, Haggard P (2008) The What, When, Whether model of intentional action. Neuroscientist 14:319–325. 10.1177/1073858408317417 18660462 10.1177/1073858408317417

[CR7] Brenner E, Smeets JB (2015) How people achieve their amazing temporal precision in interception. J Vis 15:8. 10.1167/15.3.8 25767094 10.1167/15.3.8

[CR8] Brozzoli C, Makin TR, Cardinali L, Holmes NP, Farnè A (2012) Peripersonal space: a multisensory interface for body–object interactions. In: The neural bases of multisensory processes (Murray MM and Wallace MT, ed), Chapter 23. Boca Raton (FL): CRC Press/Taylor & Francis 22593895

[CR9] Buckingham G, Donald H (2019) Move on up: fingertip forces and felt heaviness are modulated by the goal of the lift. Atten Percept Psychophys 81:2384–2390. 10.3758/s13414-019-01703-w 30949958 10.3758/s13414-019-01703-w PMC6848048

[CR10] Camponogara I (2023) The integration of action-oriented multisensory information from target and limb within the movement planning and execution. Neurosci Biobehav Rev 151:105228. 10.1016/j.neubiorev.2023.105228 37201591 10.1016/j.neubiorev.2023.105228

[CR11] Castiello U, Giordano BL, Begliomini C, Ansuini C, Grassi M (2010) When ears drive hands: the influence of contact sound on reaching to grasp. PLoS One 5:e12240. 10.1371/journal.pone.0012240 20808929 10.1371/journal.pone.0012240 PMC2923194

[CR12] Diederich A, Colonius H (2004) Bimodal and trimodal multisensory enhancement: Effects of stimulus onset and intensity on reaction time. Percept Psychophys 66:1388–1404. 10.3758/bf03195006 15813202 10.3758/bf03195006

[CR13] Elshout JA, Van der Stoep N, Nijboer TCW, Van der Stigchel S (2020) Motor congruency and multisensory integration jointly facilitate visual information processing before movement execution. Exp Brain Res 238:667–673. 10.1007/s00221-019-05714-9 32036413 10.1007/s00221-019-05714-9 PMC7080670

[CR14] Ernst MO, Banks MS (2002) Humans integrate visual and haptic information in a statistically optimal fashion. Nature 415:429–433. 10.1038/415429a 11807554 10.1038/415429a

[CR15] Flanagan JR, Bowman MC, Johansson RS (2006) Control strategies in object manipulation tasks. Curr Opin Neurobiol 16:650–659. 10.1016/j.conb.2006.10.005 17084619 10.1016/j.conb.2006.10.005

[CR16] Flash T, Hochner B (2005) Motor primitives in vertebrates and invertebrates. Curr Opin Neurobiol 15:660–6. 10.1016/j.conb.2005.10.011 16275056 10.1016/j.conb.2005.10.011

[CR17] Fogassi L, Gallese V (2004) Action as a Binding Key to Multisensory Integration. In: Calvert GA, Spence C, Stein BE (eds) The Handbook of Multisensory Processes. MIT Press, Cambridge, pp 425–441

[CR18] Georgopoulos AP (2002) Cognitive motor control: spatial and temporal aspects. Curr Opin Neurobiol 12:678–683. 10.1016/s0959-4388(02)00382-3 12490258 10.1016/s0959-4388(02)00382-3

[CR19] Graziano MS, Cooke DF (2006) Parieto-frontal interactions, personal space, and defensive behavior. Neuropsychologia 44:2621–35. 10.1016/j.neuropsychologia.2005.09.011 17128446 10.1016/j.neuropsychologia.2005.09.011

[CR20] Helbig HB, Ernst MO (2007) Optimal integration of shape information from vision and touch. Exp Brain Res 179:595–606. 10.1007/s00221-006-0814-y 17225091 10.1007/s00221-006-0814-y

[CR21] Hoffmann J, Sebald A, Stöcker C (2001) Irrelevant response effects improve serial learning in serial reaction time tasks. J Exp Psychol Learn Mem Cogn 27:470–82. 10.1037/0278-7393.27.2.470 11294444 10.1037/0278-7393.27.2.470

[CR22] Hoffstaedter F, Grefkes C, Zilles K, Eickhoff SB (2013) The “what” and “when” of self-initiated movements. Cereb Cortex 23:520–530. 10.1093/cercor/bhr391 22414772 10.1093/cercor/bhr391 PMC3593700

[CR23] Johansson RS, Flanagan JR (2009) Coding and use of tactile signals from the fingertips in object manipulation tasks. Nat Rev Neurosci 10:345–359. 10.1038/nrn2621 19352402 10.1038/nrn2621

[CR24] Johansson RS, Westling G, Bäckström A, Flanagan JR (2001) Eye-hand coordination in object manipulation. J Neurosci 21:6917–6932. 10.1523/JNEUROSCI.21-17-06917.2001 11517279 10.1523/JNEUROSCI.21-17-06917.2001 PMC6763066

[CR25] Kawato M, Wolpert D (1998) Internal models for motor control. Novartis Found Symp 218: 291–304; discussion 304–347. 10.1002/9780470515563.ch16 10.1002/9780470515563.ch16 9949827

[CR26] Land MF (2006) Eye movements and the control of actions in everyday life. Prog Retin Eye Res 25:296–324. 10.1016/j.preteyeres.2006.01.002 16516530 10.1016/j.preteyeres.2006.01.002

[CR27] Latash ML, Levin MF, Scholz JP, Schöner G (2010) Motor control theories and their applications. Medicina (Kaunas) 46:382–392 20944446 PMC3017756

[CR28] Luan M, Maurer H, Mirifar A, Beckmann J, Ehrlenspiel F (2021) Multisensory action effects facilitate the performance of motor sequences. Atten Percept Psychophys 83:475–483. 10.3758/s13414-020-02179-9 33135098 10.3758/s13414-020-02179-9 PMC7875850

[CR29] McGarity-Shipley MR, Markovik Jantz S, Johansson RS, Wolpert DM, Flanagan JR (2023) Fast feedback responses to categorical sensorimotor errors that do not indicate error magnitude are optimized based on short- and long-term memory. J Neurosci 43:8525–8535. 10.1523/JNEUROSCI.1990-22.2023 37884350 10.1523/JNEUROSCI.1990-22.2023 PMC10711696

[CR30] Meredith MA, Stein BE (1986) Visual, auditory, and somatosensory convergence on cells in superior colliculus results in multisensory integration. J Neurophysiol 56:640–662. 10.1152/jn.1986.56.3.640 3537225 10.1152/jn.1986.56.3.640

[CR31] Michalski J, Green AM, Cisek P (2020) Reaching decisions during ongoing movements. J Neurophysiol 123:1090–1102. 10.1152/jn.00613.2019 32049585 10.1152/jn.00613.2019 PMC7099481

[CR32] Miller LM, D’Esposito M (2005) Perceptual fusion and stimulus coincidence in the cross-modal integration of speech. J Neurosci 25:5884–5893. 10.1523/JNEUROSCI.0896-05.2005 15976077 10.1523/JNEUROSCI.0896-05.2005 PMC6724802

[CR33] Miller RL, Stein BE, Rowland BA (2017) Multisensory integration uses a real-time unisensory-multisensory transform. J Neurosci 37:5183–5194. 10.1523/JNEUROSCI.2767-16.2017 28450539 10.1523/JNEUROSCI.2767-16.2017 PMC5444199

[CR34] Oostwoud Wijdenes L, Brenner E, Smeets JB (2014) Analysis of methods to determine the latency of online movement adjustments. Behav Res Methods 46:131–139. 10.3758/s13428-013-0349-7 23637021 10.3758/s13428-013-0349-7

[CR35] Ortega L, Guzman-Martinez E, Grabowecky M, Suzuki S (2014) Audition dominates vision in duration perception irrespective of salience, attention, and temporal discriminability. Atten Percept Psychophys 76:1485–1502. 10.3758/s13414-014-0663-x 24806403 10.3758/s13414-014-0663-x PMC4096074

[CR36] Pettypiece CE, Goodale MA, Culham JC (2010) Integration of haptic and visual size cues in perception and action revealed through cross-modal conflict. Exp Brain Res 201:863–873. 10.1007/s00221-009-2101-1 19949777 10.1007/s00221-009-2101-1

[CR37] Roy C, Dalla Bella S, Lagarde J (2017) To bridge or not to bridge the multisensory time gap: bimanual coordination to sound and touch with temporal lags. Exp Brain Res 235:135–151. 10.1007/s00221-016-4776-4 27655357 10.1007/s00221-016-4776-4

[CR38] Sabes PN (2011) Sensory integration for reaching: models of optimality in the context of behavior and the underlying neural circuits. Prog Brain Res 191:195–209. 10.1016/B978-0-444-53752-2.00004-7 21741553 10.1016/B978-0-444-53752-2.00004-7 PMC3361512

[CR39] Säfström D, Edin BB (2004) Task requirements influence sensory integration during grasping in humans. Learn Mem 11:356–363. 10.1101/lm.71804 15169866 10.1101/lm.71804 PMC419739

[CR40] Säfström D, Edin BB (2008) Prediction of object contact during grasping. Exp Brain Res 190:265–277. 10.1007/s00221-008-1469-7 18592227 10.1007/s00221-008-1469-7

[CR41] Säfström D, Flanagan JR, Johansson RS (2013) Skill learning involves optimizing the linking of action phases. J Neurophysiol 110:1291–1300. 10.1152/jn.00019.2013 23741046 10.1152/jn.00019.2013

[CR42] Säfström D, Johansson RS, Flanagan JR (2014) Gaze behavior when learning to link sequential action phases in a manual task. J Vis 14:3. 10.1167/14.4.3 24695992 10.1167/14.4.3

[CR43] Sailer U, Flanagan JR, Johansson RS (2005) Eye-hand coordination during learning of a novel visuomotor task. J Neurosci 25:8833–8842. 10.1523/JNEUROSCI.2658-05.2005 16192373 10.1523/JNEUROSCI.2658-05.2005 PMC6725583

[CR44] Serino A (2019) Peripersonal space (PPS) as a multisensory interface between the individual and the environment, defining the space of the self. Neurosci Biobehav Rev 99:138–159. 10.1016/j.neubiorev.2019.01.016 30685486 10.1016/j.neubiorev.2019.01.016

[CR45] Shadmehr R, Smith MA, Krakauer JW (2010) Error correction, sensory prediction, and adaptation in motor control. Annu Rev Neurosci 33:89–108. 10.1146/annurev-neuro-060909-153135 20367317 10.1146/annurev-neuro-060909-153135

[CR46] Sobinov AR, Bensmaia SJ (2021) The neural mechanisms of manual dexterity. Nat Rev Neurosci 12:741–757. 10.1038/s41583-021-00528-7 10.1038/s41583-021-00528-7 PMC9169115 34711956

[CR47] Spence C, Nicholls M, Driver J (2001) The cost of expecting events in the wrong sensory modality. Percept Psychophys 63:330–336. 10.3758/bf03194473 11281107 10.3758/bf03194473

[CR48] Stefanescu MR, Thürling M, Maderwald S, Wiestler T, Ladd ME, Diedrichsen J, Timmann D (2013) A 7T fMRI study of cerebellar activation in sequential finger movement tasks. Exp Brain Res 228:243–254. 10.1007/s00221-013-3558-5 23732948 10.1007/s00221-013-3558-5

[CR49] Stein BE (1998) Neural mechanisms for synthesizing sensory information and producing adaptive behaviors. Exp Brain Res 123:124–135. 10.1007/s002210050553 9835401 10.1007/s002210050553

[CR50] Stein BE, Stanford TR, Ramachandran R, Perrault TJ Jr, Rowland BA (2009) Challenges in quantifying multisensory integration: alternative criteria, models, and inverse effectiveness. Exp Brain Res 198:113–126. 10.1007/s00221-009-1880-8 19551377 10.1007/s00221-009-1880-8 PMC3056521

[CR51] Stöcker C, Hoffmann J (2004) The ideomotor principle and motor sequence acquisition: tone effects facilitate movement chunking. Psychol Res 68:126–37. 10.1007/s00426-003-0150-9 14648225 10.1007/s00426-003-0150-9

[CR52] Stöcker C, Sebald A, Hoffmann J (2003) The influence of response–effect compatibility in a serial reaction time task. Q J Exp Psychol A 56:685–703. 10.1080/02724980244000585 12745836 10.1080/02724980244000585

[CR02] Turvey MT, Sheya A (2017) Non-obvious influences on perception-action abilities. Psychon Bull Rev 24:1597–1603. 10.3758/s13423-016-1223-2 28188562 10.3758/s13423-016-1223-2

[CR53] van Polanen V (2022) Multisensory information about changing object properties can be used to quickly correct predictive force scaling for object lifting. Exp Brain Res 240:2121–2133. 10.1007/s00221-022-06404-9 35786747 10.1007/s00221-022-06404-9

[CR54] van Dijk L, van der Sluis C, Bongers RM (2017) Reductive and Emergent Views on Motor Learning in Rehabilitation Practice. J Mot Behav 49:244–254. 10.1080/00222895.2016.1191418 27592838 10.1080/00222895.2016.1191418

[CR55] van Polanen V, Tibold R, Nuruki A, Davare M (2019) Visual delay affects force scaling and weight perception during object lifting in virtual reality. J Neurophysiol 121:1398–1409. 10.1152/jn.00396.2018 30673365 10.1152/jn.00396.2018 PMC6485735

[CR56] Whitwell RL, Lambert LM, Goodale MA (2008) Grasping future events: explicit knowledge of the availability of visual feedback fails to reliably influence prehension. Exp Brain Res 188:603–11. 10.1007/s00221-008-1395-8 18443765 10.1007/s00221-008-1395-8

[CR57] Wiestler T, Diedrichsen J (2013) Skill learning strengthens cortical representations of motor sequences. Elife 2:e00801. 10.7554/eLife.00801 23853714 10.7554/eLife.00801 PMC3707182

[CR58] Wolpert DM, Flanagan JR (2016) Computations underlying sensorimotor learning. Curr Opin Neurobiol. 10.1016/j.conb.2015.12.003 26719992 10.1016/j.conb.2015.12.003 PMC6103431

[CR59] Yang F, Feldman AG (2010) Reach-to-grasp movement as a minimization process. Exp Brain Res 201:75–92. 10.1007/s00221-009-2012-1 19771417 10.1007/s00221-009-2012-1

[CR01] Zhang L, Guberman S, Feldman AG (2022) Shifts in the eye-centered frame of reference may underlie saccades, visual perception, and eye-hand coordination. J Neurophysiol 128:1025–1039. 10.1152/jn.00531.2021 36070246 10.1152/jn.00531.2021

